# Predicting Aboveground Carbon Storage in Different Types of Forests in South Subtropical Regions Using Machine Learning Models

**DOI:** 10.1002/ece3.71499

**Published:** 2025-05-26

**Authors:** Jiarun Liu, Zihang Yang, Lin Li, Xiaoxue Chu, Shiguang Wei, Juyu Lian

**Affiliations:** ^1^ School of Life & Environmental Sciences Guilin University of Electronic Technology Guilin Guangxi China; ^2^ Key Laboratory of Ecology of Rare and Endangered Species and Environmental Protection, Ministry of Education – Guangxi Key Laboratory of Landscape Resources Conservation and Sustainable Utilization in Lijiang River Basin Guangxi Normal University Guilin Guangxi China; ^3^ Key Laboratory of National Forestry and Grassland Administration on Plant Conservation and Utilization in Southern China, South China Botanical Garden Chinese Academy of Sciences Guangzhou China; ^4^ Guangdong Provincial Key Laboratory of Applied Botany, South China Botanical Garden Chinese Academy of Sciences Guangzhou China

**Keywords:** aboveground carbon storage prediction, factor contributions, machine learning models, multi‐layer perceptron, random forest, SHAP values, south subtropical evergreen broad‐leaved forest, support vector machine, XGBoost model

## Abstract

Motivated by the need to enhance the accuracy of forest aboveground carbon storage (ACS) assessments, this study aimed to explore the effectiveness of different machine learning models in predicting ACS across various subtropical forest types in southern China. The study was conducted in southern China, focusing on different types of subtropical forests. This region harbors several types of subtropical forests, which are rarely found at similar latitudes in the world. Variance inflation factor was employed to screen independent variables, resulting in the selection of 13 significant predictors. Four machine learning models—support vector machine (SVM), random forest (RF), multi‐layer perceptron (MLP), and extreme gradient boosting (XGB)—were constructed to estimate carbon storage. Model performance was evaluated using root mean square error, coefficient of determination (*R* ^2^), and mean absolute error. The model with the best generalization ability was selected to calculate SHAP values for each predictor. The XGB model demonstrated superior performance across all forest types, with *R* ^2^ values ranging from 0.898 to 0.974. In mountainous evergreen broad‐leaved forests, the prediction accuracy followed the order of XGB>MLP>SVM>RF. In valley rainforests, MLP showed the highest *R* ^2^ value, but with higher MAE and RMSE, making it the second‐best choice. The RF model performed moderately, while the SVM model showed the poorest performance. The SHAP values indicated that maximum diameter at breast height, slope, mean DBH, species evenness, altitude, and maximum tree height had significant effects on ACS. XGB model exhibits the best prediction performance and strongest adaptability for estimating ACS in subtropical southern China forests. Additionally, the MLP model can serve as an effective model for assessing carbon storage in valley rainforests within this region. Machine learning methods provide valuable references for predicting and assessing ACS in different types of zonal forests.

## Graphical abstract

*Multi‐model comparison*: Study compared SVM, RF, MLP, and XGB models for forest carbon stock assessment, finding XGB superior across all forest types. *XGB model performance*: XGB model achieved highest accuracy (*R* ^2^ = 0.898–0.974) and lowest errors (RMSE, MAE) in all forest types. *MLP advantage in Valley Rainforest*: MLP showed highest *R* ^2^ in valley rainforest, though inferior to XGB overall. *Key impact factors*: The SHAP analysis identified the following key impact factors: maximum DBH (DBHmax) and slope had positive impacts, while average DBH (DBHmean), species evenness, elevation, and maximum tree height had negative impacts.

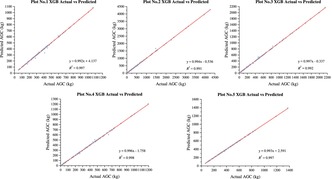

## 1 Introduction

The assessment and prediction of forest aboveground carbon storage (ACS) contribute significantly to a deeper understanding of the carbon sink function of forests. Forest plants absorb CO_2_ from the atmosphere and store it in the form of biomass. Forests are the cornerstone of terrestrial ecosystems and constitute the largest carbon pool, accounting for 90% of terrestrial vegetation biomass (Kozlowski and Song 2022). Forests function as highly efficient natural carbon sequestration systems (Tang et al. 2018). Machine learning models provide a valuable tool for improving carbon storage predictions and optimizing forest management.

Currently, machine learning models such as Support Vector Machines (SVM), Random Forests (RF), Multi‐Layer Perceptrons (MLP), and Extreme Gradient Boosting (XGB) have been effectively applied in various fields (da Rocha et al. 2023). Among them, the SVM model is one of the most widely used supervised machine learning algorithms. It can effectively solve nonlinear problems, efficiently analyze classification and regression problems, and provide highly interpretable results. Therefore, it is widely used in ecosystem research (Ghannam and Techtmann 2021).

Random Forest (RF) model is an ensemble learning method that combines multiple decision trees, enabling it to effectively tackle both regression and classification problems (Prasad et al. 2006), thereby increasing the accuracy of the model. The MLP model, a type of deep learning model, is a feedforward neural network with multiple layers of neurons (Singh et al. 2017). Consequently, it can process complex data and automatically extract critical features (Zhang et al. 2018), making it adept at dealing with the diverse and dynamic environments of forests.

XGBoost (XGB) model is a powerful machine learning algorithm that is particularly suitable for solving classification and regression problems. It uses decision trees as its basic learner, iteratively training a series of decision trees and summing their predictions with weights to improve the performance of the model (Chen et al. 2016).

The rapid development of machine learning and optimization algorithms has provided new methodologies to accurately estimate forest carbon storage at various scales (Thanh et al. 2024). While the field‐based measurement and modeling approaches have been widely used for assessing forest vegetation carbon storage (Sun and Liu 2019), they require detailed field survey data to estimate carbon storage, which limits their applicability at large spatial and temporal scales. By constructing and optimizing high‐performance deep learning algorithms and models, machine learning models with higher degrees of adaptability for forest carbon storage assessment and prediction can be obtained (da Rocha et al. 2023), effectively enhancing the accuracy of aboveground forest carbon storage estimation and prediction.

Dantas et al. used support vector machines SVM and MLP models to predict carbon storage in tropical forests in southeastern Brazil. Their results showed that both SVM and MLP models performed well, with the MLP model demonstrating higher prediction accuracy (Dantas et al. 2021). In recent years, the application of machine learning in forest carbon storage prediction has become increasingly prevalent. To improve model prediction performance, remote‐sensing vegetation indices have been introduced as modeling parameters (Cheng et al. 2024). Additionally, forest species diversity, which affects the distribution of forest ACS, can also serve as a screening parameter for machine learning models.

The prediction accuracy of machine learning models is crucial for their selection and widespread application. During the process of using machine learning to predict forest ACS, collinearity relationships among various variables (biological and non‐biological factors influencing carbon storage accumulation and distribution) can potentially increase errors in model training results (Shaheen et al. 2023). Employing a stepwise selection model with Variance Inflation Factor (VIF) as the evaluation criterion can exclude collinear variables during each iteration, helping to avoid collinearity issues in model training variables (O'Brien 2007). This approach can effectively enhance the prediction accuracy of forest carbon storage.

China's subtropical forests in the southern regions are rarely found at similar latitudes worldwide and exhibit a transitional character from tropical to subtropical with complex community structures and rich species diversity, making them significant contributors to forest carbon sinks (Zhou et al. 2006; Njoroge et al. 2021). Under climate change perturbations, the structural dynamics of these subtropical forests in southern China have undergone notable changes (Wei et al. 2024), necessitating precise assessments of their carbon storage.

This study, based on field monitoring data from different types of subtropical forest plots in Dinghushan, Guangdong Province, China, aims to screen biological and non‐biological factors that significantly influence carbon storage. Four different machine learning models—SVM, RF, MLP, and XGB—each with their unique performance characteristics, were constructed to predict carbon storage in different subtropical forest communities. The predictive performance of these models was compared across different forest plot types to identify the model with the strongest generalization ability. Additionally, the study quantifies the contribution of various influencing factors to the accumulation and distribution of carbon storage in subtropical forests, facilitating effective prediction of the regional forest ACS distribution. This methodology also provides a reference for accurately assessing ACS in other zonal forests.

## 2 Materials and Methods

### 2.1 Overview of the Study Area

**FIGURE 1:**
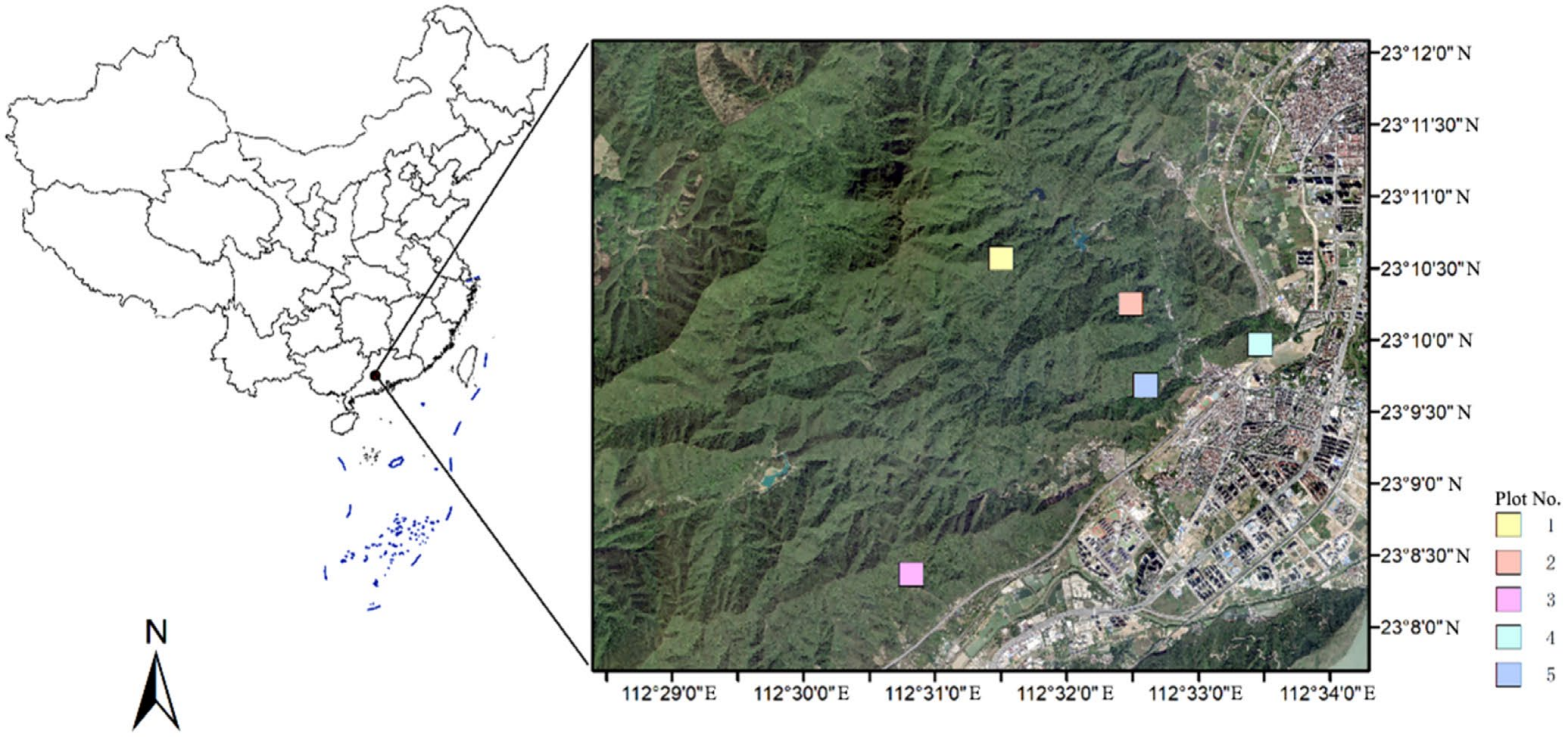
Location map of the five 1 hm^2^ sample plots.

In this study, five 1 hm^2^ plots of different types of forest communities located within the Dinghushan National Nature Reserve in Guangdong Province, China, were selected as the research sites (Figure 1). The Dinghushan National Nature Reserve (112°30′39″–112°33′41″ E, 23°09′21″–23°11′30″ N) is characterized by a subtropical monsoon climate, with mountainous and hilly terrain. This region harbors several types of subtropical forests, which are rarely found at similar latitudes in the world. The annual average temperature is 20.9°C, with a monthly average of 12.6°C in January and 28.0°C in July. The average annual precipitation is 1929 mm, with most rainfall occurring from April to September. The average annual evaporation is 1115 mm, and the relative humidity is 82% (Li et al. 2009).

### 2.2 Plot Investigation and ACS Statistics

The five 1 hm^2^ (1 ha) plots of different subtropical forest types at Dinghushan, each established according to the survey technical specifications of the Center for Tropical Forest Science (CTFS) at the Smithsonian Tropical Research Institute in the United States, are field surveyed every five years. During these surveys, detailed information is recorded for all individual plants with a diameter at breast height (DBH) of 1 cm or greater. This information includes the species name, DBH, plant coordinates, tree height, and habitat details. The Dinghushan forest plots contain a diverse range of vegetation types typical of subtropical mountainous regions (Table 1).

**TABLE 1:** Basic data of the forest sample plot in Dinghushan.

Plot no.	Plant community type	Elevation (m)	Species count	Individual count	ACS (kg)
1	Mountain broadleaf forests	602–660	90	4050	36,896
2	Valley rainforests	90–133	88	1997	107,657
3	Evergreen broadleaf forests	182–256	96	4129	49,556
4	Evergreen coniferous forests	38–93	61	2138	36,786
5	Mixed coniferous‐broadleaf forests	50–85	60	3031	42,701

Given that the average annual precipitation in Dinghushan from 2005 to 2020 was 1929 mm, exceeding the threshold of 1500 mm for classifying a forest as wet, we employed a wet forest aboveground biomass model (Chave et al. 2005) to predict the aboveground biomass of each individual in the sample plots. This model applies to all species within the study area. The actual carbon storage of each species was calculated using the predicted biomass in conjunction with the measured wood density (WD) of the species. The specific calculation formula is as follows (Chave et al. 2005; Shen et al. 2016):

(1) $$\begin{array}{c} \mathrm{AGB}=\\ \mathrm{WD}\times \exp\left(-1.499+2.148\ln\mathrm{DBH}+0.207{\left(\ln\mathrm{DBH}\right)}^{2}-0.0281{\left(\ln\mathrm{DBH}\right)}^{3}\right) \end{array}$$

(2) ACS=AGB×0.46

WD is the wood density of the species, measured in grams per cubic centimeter (g/cm^3^). DBH stands for the diameter at breast height of the tree, measured in centimeters (cm). AGB denotes the aboveground biomass, measured in kilograms (kg). ACS is the aboveground carbon storage, also measured in kilograms (kg). This study focuses on the aboveground carbon storage of species within a unit area of 10 m × 10 m.

### 2.3 Factor Selection

During the model‐building process, if there is a high degree of correlation between environmental factors, this can lead to the problem of multicollinearity, which affects the accuracy of model parameter estimation. To address this, we employ a stepwise regression analysis method in conjunction with the variance inflation factor (VIF) to screen the species characteristics, diversity, environmental factors, and remote sensing data (Table S1) that influence carbon storage. A linear equation is constructed with 21 factors as independent variables, and the VIF values are calculated for these 21 independent variables. First, the variable with the highest VIF value is selected and removed. Then, a new linear equation is established with the remaining 20 variables and an arbitrary constant, and new VIF values are computed. The variable with the highest VIF value is removed again. This process is repeated until the VIF values of all remaining variables in the linear equation are less than 10, at which point the screening process is terminated. The formula for calculating the VIF is as follows (O'Brien 2007):

(3) $$\mathrm{VIF}=\frac{1}{1-R^{2}}$$

*R* ^2^, also known as the coefficient of determination, is calculated using the formula:

(4) $$R^{2}=1-\left|\frac{\mathrm{SSR}}{\mathrm{SST}}\right|$$

where *SSR* is the Sum of Squares of Residuals, and *SST* is the Total Sum of Squares.

**FIGURE 2:**
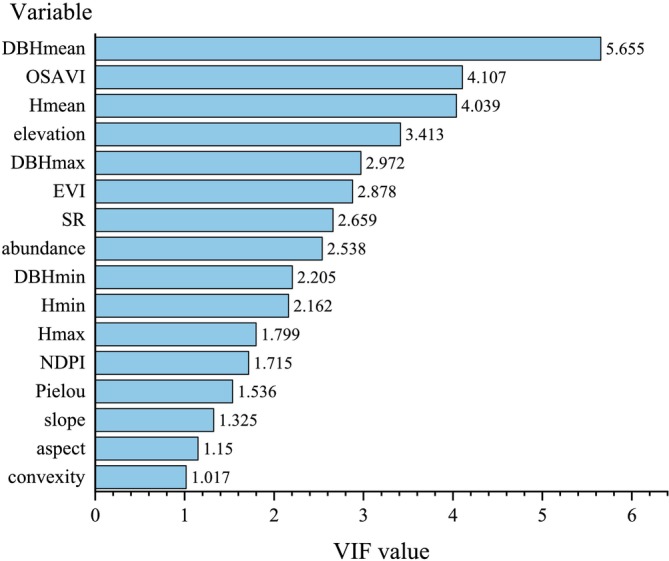
Effective parameters with VIF values all less than 10.

In machine learning prediction, increasing the number of factors (independent variables) allows for a more comprehensive consideration of the factors influencing carbon storage, but it also increases the complexity of the prediction model and the likelihood of overfitting. Therefore, we use VIF (Variance Inflation Factor) to select the most appropriate input independent variables to establish a model that not only improves the fitting performance but also reduces the potential for overfitting. The final effective parameters selected for model training are DBHmean, OSAVI, Hmean, elevation, DBHmax, EVI, SR, tree abundance, DBHmin, Hmin, Hmax, NDPI, slope, aspect, and convexity (Figure 2).

### 2.4 Model Selection and Evaluation

Based on the measured values of each impact factor, four machine learning models with different performances (SVM, RF, MLP, and XGB) were constructed (Table 2) to fit different types of forest ACS. SVM and MLP are non‐explainable models ideal for capturing complex nonlinear relationships in prediction tasks, while RF and XGB are interpretable models offering feature importance and explainability, aiding in identifying key ecological drivers. The models were tested by using a 10‐fold cross‐validation method, and the monitoring data of ACS in sample plots were randomly divided into a 70% training data set and a 30% test data set. Cross‐validation assessed model performance and used validation errors to fine‐tune parameters. It involved splitting the dataset into subsets, repeatedly training and validating the model, analyzing performance variations, and refining parameters based on feedback.

**TABLE 2:** Introduction to four models.

Model	Description	Applications in ecology
SVM	It is used for supervised learning, primarily for classification, by constructing a hyperplane in a high‐dimensional space that optimally separates data of different categories (Ghannam and Techtmann 2021)	Predicting ACS (Dantas et al. 2021)
RF	Ensemble learning method, using decision trees as base learners, constructs multiple base learners through random resampling and finally combines their prediction results (Prasad et al. 2006)	Simulates and identifies key factors affecting soil organic carbon content (Shen et al. 2023)
MLP	A feedforward neural network, consisting of an input layer, hidden layers, and an output layer, learns and makes predictions through the forward propagation and backpropagation algorithms (Singh et al. 2017)	Predicting ACS (Dantas et al. 2021)
XGB	A powerful machine learning algorithm, particularly suitable for classification and regression problems, utilizes decision trees as base learners and iteratively improves model performance (Chen et al. 2016)	Estimating aboveground biomass (Thanh et al. 2024)

The Root mean square error (RMSE), the mean relative error (MAE), and the coefficient of determination (*R* ^2^) were used to evaluate the goodness of the model. The lower the RMSE and MAE, the better the predictive power of the model, and the higher the *R* ^2^, the higher the goodness of the fit. Compare the above three indexes of the training set and the test set at the same time to eliminate the risk of overfitting. The calculation formulas are as follows:

(5) $$\text{RMSE}=\sqrt{\frac{\sum_{i=1}^{n}\left(y_{i}-{\hat{y}}_{i}\right)}{n}}$$

(6) $$\mathrm{MAE}=\frac{\sum_{i=1}^{n}|y_{i}-{\hat{y}}_{i}|}{n}$$

where *y* is the observed value, *ŷ* is the predicted value by the model, *i* is the sample index, and *n* is the number of samples.

### 2.5 Data Processing

The research data processing is running on the R platform (R Core Team 2020 (version 4.2.3). R: A language and environment for statistical computing. R Foundation for Statistical Computing, Vienna, Austria: https://www.R‐project.org/). VIF variable filtering is computed programmatically using the “car” software package. The K‐fold test is calculated by the caret software package. The SVM model was programmed and calculated by the “e1071” software package. The RF model was programmed and calculated using the software package “randomForest”. The XGB model is computed programmatically using the “xgboost” software package. The multi‐layer perceptron MLP is programmed and calculated using the “nnet” software package. The SHAP value is calculated using the shapviz package.

## 3 Results

### 3.1 Model Prediction Results

**FIGURE 3:**
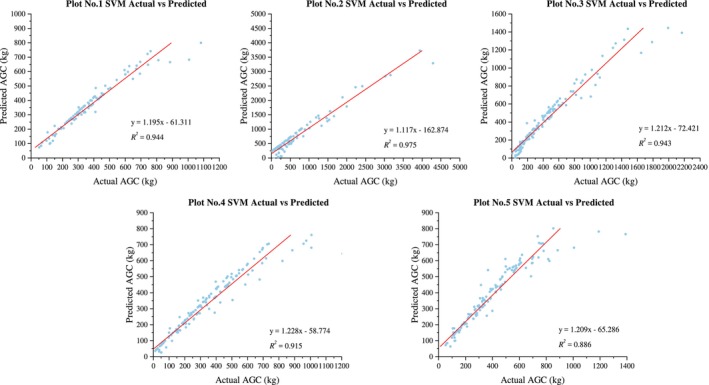
Prediction results of SVM model.

The evaluation results (Figure 3) indicate that the SVM model exhibits a satisfactory predictive performance for the ACS of five different forest types in the southern subtropical region, with all *R* ^2^ values exceeding 0.55. The goodness of fit, ranked from highest to lowest, is as follows: montane evergreen broad‐leaved forest (0.718) > evergreen broad‐leaved forest (0.682) > evergreen coniferous forest (0.634) > mixed coniferous and broad‐leaved forest (0.630) > valley rainforest (0.579).

**FIGURE 4:**
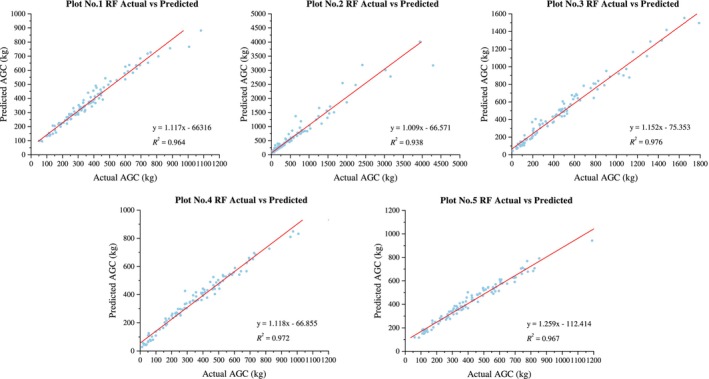
Prediction results of RF model.

The RF model also demonstrates good predictive performance for the ACS of five different types of forests in the southern subtropical region, with all *R* ^2^ values above 0.6 (Figure 4). The goodness of fit, ranked from highest to lowest, is as follows: evergreen broad‐leaved forest (0.782) > evergreen coniferous forest (0.716) > valley rainforest (0.712) > montane evergreen broad‐leaved forest (0.700) > mixed coniferous and broad‐leaved forest (0.637).

**FIGURE 5:**
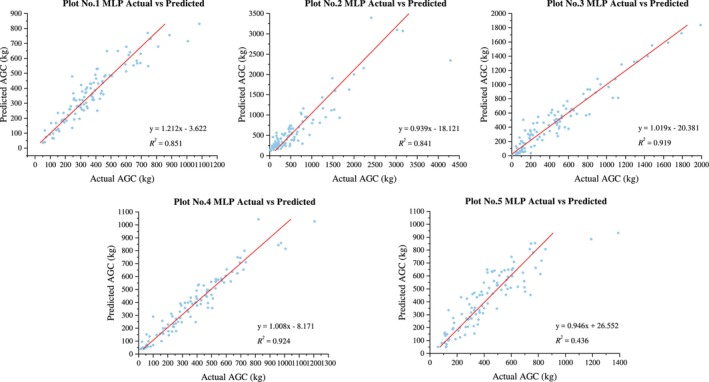
Prediction results of MLP model.

The MLP model exhibits excellent predictive performance for the ACS of five different forest types in the southern subtropical region, with all *R* ^2^ values exceeding 0.75 (Figure 5). The goodness of fit, ranked from highest to lowest, is as follows: evergreen coniferous forest (0.924) > valley rainforest (0.923) > evergreen broad‐leaved forest (0.919) > montane evergreen broad‐leaved forest (0.847) > mixed coniferous and broad‐leaved forest (0.767).

**FIGURE 6:**
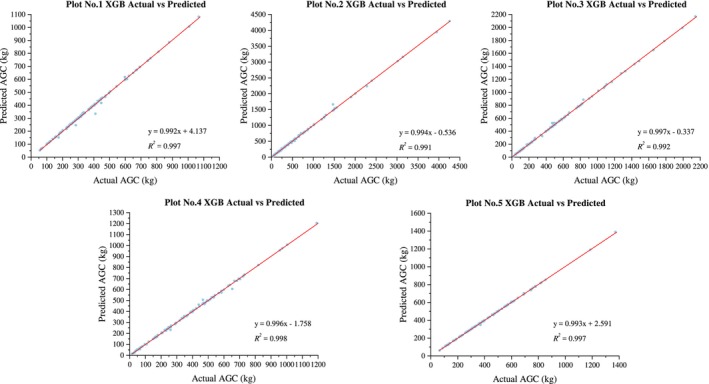
Prediction results of XGB model.

The XGBoost model demonstrates strong predictive performance for the ACS of five different forest types in the southern subtropical region, with all *R* ^2^ values exceeding or approaching 0.9 (Figure 6), and conducted an effective assessment of the risk of overfitting. The goodness of fit, ranked from highest to lowest, is as follows: evergreen coniferous forest (0.981) > montane evergreen broad‐leaved forest (0.979) > evergreen broad‐leaved forest (0.975) > mixed coniferous and broad‐leaved forest (0.971) > valley rainforest (0.899).

**FIGURE 7:**
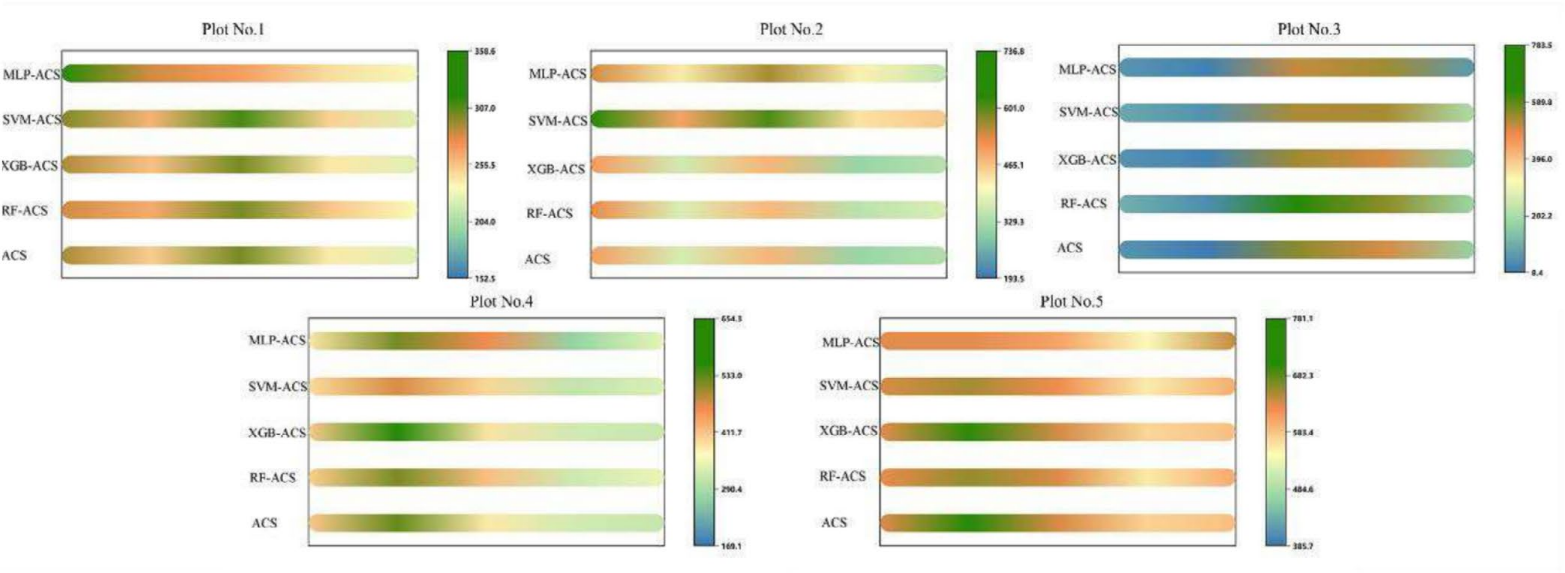
Comparison of the predicted and actual values of ACS by different models across five plots.

Figure 7 presents a comparison of the predicted and actual values of ACS by the XGB, SVM, RF, and MLP models across five sample plots. Specifically, in mountain broadleaf forests (Plot No. 1), the predicted values are primarily concentrated within the [255, 307] range, with fewer values distributed in the [307, 358.6] range, which is in good agreement with the distribution of the actual values. In valley rainforests (Plot No. 2), both the predicted and actual values are mainly distributed across the three ranges of [8.4, 202.2], [202.2, 396.6], and [396.6, 783.5]. In evergreen broadleaf forests (Plot No. 3), the predicted and actual values are concentrated within the two ranges of [329.3, 465.1] and [465.1, 601.1]. In evergreen coniferous forests (Plot No. 4), the predicted and actual values are mainly distributed within the two ranges of [290.4, 411.7] and [411.7, 533.0]. Finally, in mixed coniferous‐broadleaf forests (Plot No. 5), the predicted and actual values are concentrated within the range [583.4, 682.3].

### 3.2 Results of Model Evaluation

**FIGURE 8:**
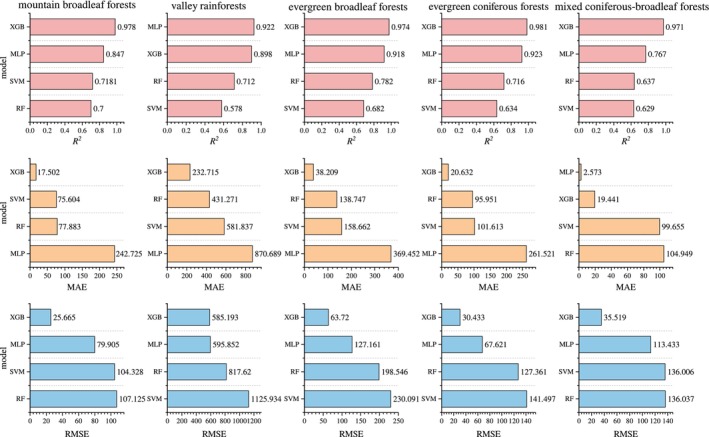
Evaluation metrics of various models.

A comprehensive comparison of *R* ^2^, MAE, and RMSE values of the models in different forest types (Figure 8) reveals that within the mountainous evergreen broad‐leaved forests, the XGB model achieves the best performance in terms of *R* ^2^, MAE, and RMSE. The MLP model performs well in *R* ^2^ and RMSE but exhibits weaker performance in MAE. The SVM and RF models show moderate performance. For valley rainforest predictions, although the XGB and MLP models are similar in RMSE, the XGB model outperforms the MLP model in both MAE and *R* ^2^. Additionally, the RF model performs best in MAE.

In the evergreen broad‐leaved forests, the XGB model once again achieves the best performance across all three metrics: MAE, RMSE, and *R* ^2^ with a particularly high *R* ^2^ value of 0.974, indicating an extremely high prediction accuracy. The MLP model performs relatively well in terms of RMSE but lags behind the XGB model in terms of both MAE and *R* ^2^. The RF and SVM models show moderate performance.

For predictions in evergreen coniferous forests, the XGB model once again demonstrates its superiority, achieving the best results in MAE, RMSE, and *R* ^2^. The MLP model performs well in *R* ^2^ and RMSE but exhibits weaker performance in MAE. The SVM and RF models maintain moderate performance, while the other three models fall short in comparison.

Similarly, in mixed coniferous‐broadleaved forests, the XGB model attains the best performance across MAE, RMSE, and *R* ^2^. The MLP model performs relatively well in RMSE but falls short of the XGB model in both MAE and *R* ^2^.

In summary, the XGB model consistently demonstrates superior performance in all forest type predictions, achieving excellent results in terms of MAE, RMSE, and *R* ^2^, making it the most suitable machine learning model for predicting carbon storage in the subtropical forests of Dinghushan Mountain. Although the MLP model has a higher *R* ^2^ value than the XGB model in valley rainforest predictions, its higher MAE and RMSE values suggest that it can serve as a second‐best option for ACS predictions in such forests. The RF model performs moderately well in prediction, but is generally inferior to the XGB and MLP models. The SVM model performs poorly in terms of *R* ^2^ and RMSE.

### 3.3 Factor Contribution Analysis

**FIGURE 9:**
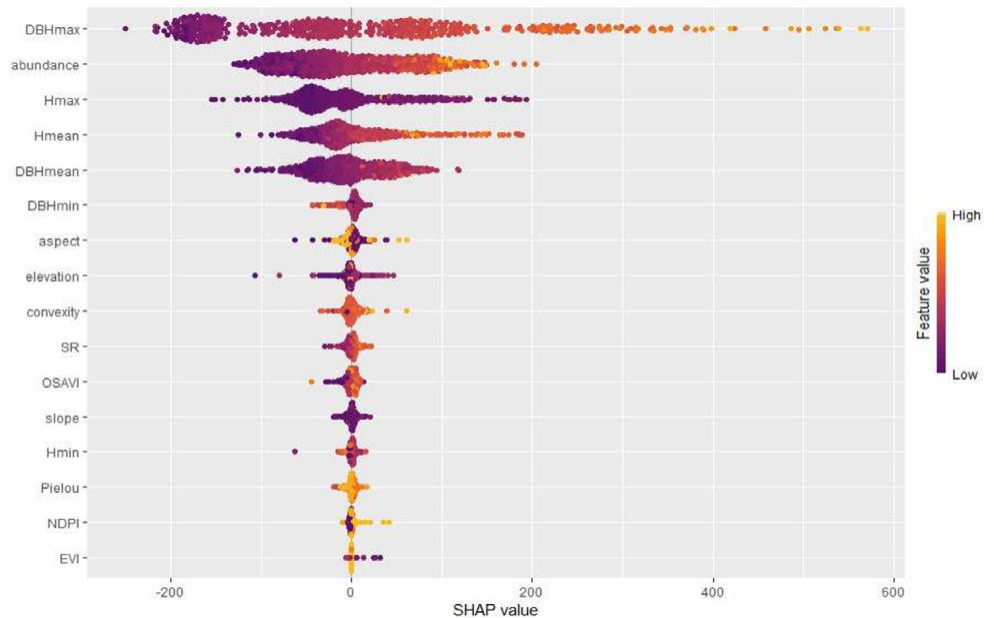
Scatter plot of contributions to model predictions (The X‐axis represents the magnitude of the SHAP values for each feature, while the Y‐axis indicates the input features. The color gradient, ranging from purple to yellow, signifies the increasing values of each feature across the points).

**FIGURE 10:**
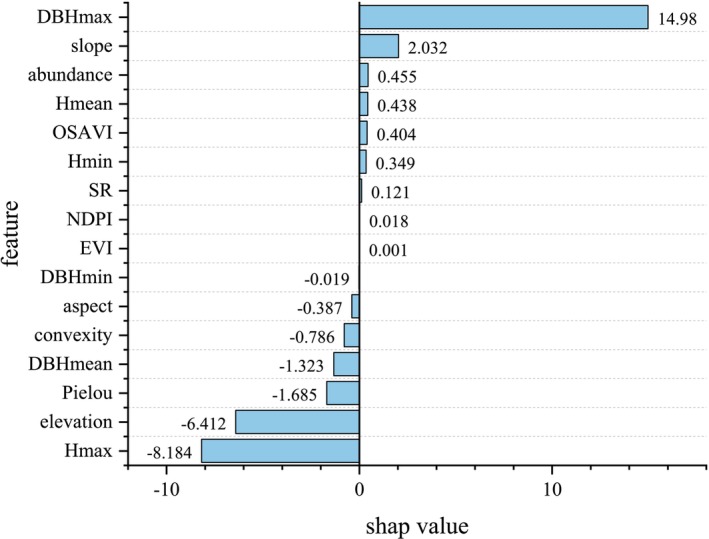
Average SHAP values of variables.

To gain a deeper understanding of the influence of different factors on the prediction of ACS in subtropical forests during the machine learning model prediction process, we utilized the XGB model, which was previously identified as having the best generalization ability, to conduct a SHAP value analysis. This analysis quantifies the contribution of each feature factor to the model's prediction results (Figures 9 and 10). The results indicate that the DBHmax and slope have significant positive effects on carbon storage. Meanwhile, features such as Hmean, tree abundance, and OSAVI have slightly positive influences on the prediction of ACS, with SHAP values of 0.438, 0.455, and 0.404, respectively.

The SHAP values for the NDPI and EVI are 0.018 and 0.001, respectively, indicating relatively small contributions but still exhibiting positive effects. However, Hmax, elevation, Pielou's species evenness, and DBHmean have significant negative impacts on the model's prediction of ACS. Specifically, DBHmean, convexity, and DBHmin have slight to minor negative effects on ACS prediction, with SHAP values of −1.323, −0.786, and −0.019, respectively.

Figure 9 is a summary plot of the SHAP values for each feature. It can be observed that when the DBHmax, tree abundance, and Hmean increase, their SHAP values also increase, indicating that these variables have a significant positive effect on ACS. In contrast, maximum tree height and elevation have negative impacts on ACS.

## 4 Discussion

### 4.1 Prediction Model for Carbon Storage in South Subtropical Forests

Our study examined the performance of various machine learning models across different forest types in the south subtropical region of China. Among the four models for predicting carbon storage in south subtropical forests, the XGB model consistently produced the most accurate predictions, demonstrating outstanding performance across all five different forest types with the highest *R* ^2^ values. This suggests that the XGB model has the highest universality and reliability for predicting carbon storage in south subtropical forests. Therefore, the XGB model can be considered the preferred choice for ACS prediction in this region. The MLP model follows closely, but while it has a high goodness of fit, its prediction accuracy requires improvement. The SVM and RF models performed similarly, with *R* ^2^ values ranging between 0.5 and 0.75.

The MLP model showed excellent performance in the evergreen broadleaf forest, with low RMSE and MAE values and a high *R* ^2^. Specifically, the MLP model achieved the highest *R* ^2^ value in predicting ACS in valley rainforests, making it potentially the best choice for such predictions. It also performed well in mountain broadleaf forests and evergreen coniferous forests. However, for mixed coniferous‐broadleaf forests, the MLP model's performance was average in terms of RMSE and *R* ^2^, with a relatively high MAE. This is probably because artificial neural networks cannot identify the relative importance and influence of individual environmental variables (Shen et al. 2023); the learning process of MLP cannot be observed within a black box, which may contribute to difficult‐to‐interpret output errors.

The SVM model demonstrated outstanding prediction results in mountain broadleaf forests and evergreen broadleaf forests, with low RMSE and high *R* ^2^ values, indicating its good predictive adaptability in these two forest types. However, in valley rainforests, evergreen coniferous forests, and mixed coniferous‐broadleaf forests, the prediction results of the SVM model were relatively poor. The reasons for this are as follows: While the SVM model can model non‐linear decision boundaries and is effective in combating overfitting, when dealing with larger datasets, the model relies solely on past records as support vectors. If there are inconsistencies in the previous data, it may lead to poor extrapolation performance of the model (Liu et al. 2018). Additionally, the SVM model is highly sensitive to parameter settings and the choice of kernel functions, with different parameters and kernel functions potentially leading to entirely different results (Gunn 1998). Therefore, for predictions in different forest types, selecting the appropriate parameters and kernel functions is crucial to ensure optimal SVM performance and accurate ACS predictions across various forest types.

RF model demonstrated exceptional performance in predicting ACS in valley rainforests, achieving the highest *R* ^2^ value and relatively good predictions for montane broadleaf forests, evergreen broadleaf forests, and coniferous forests. However, its performance was weaker in mixed coniferous‐broadleaf forests. This suggests that the RF model may have better predictive capabilities for evergreen and broadleaf forest types, while more model parameter adjustments may be required when predicting ACS in mixed coniferous‐broadleaf forests.

### 4.2 Influencing Factors of Carbon Storage in South Subtropical Forests

Tree species characteristics play a crucial role in the accumulation and distribution of their biomass carbon storage. In the subtropical forests of southern China, we found that several biological characteristic factors, including DBH, tree height, tree abundance, and the species distribution evenness, impact the distribution of ACS. Among them, DBH has the greatest influence, followed by tree height, species evenness, and abundance.

Firstly, when considering DBH as an influencing factor, the DBHmax has the greatest impact, with a SHAP value as high as 14.980, significantly contributing positively to the ACS. This finding supports the notion that large trees often serve as the primary carriers of forest ACS due to their immense biomass (Xu et al. 2015). In contrast, the DBHmean and DBHmin have SHAP values of −1.323 and −0.019, respectively, both of which have negative effects on carbon storage. This reflects the complexity of DBH distribution resulting from species composition, stand structure, and competitive relationships within the forest ecosystem. In terms of statistical area, an increase in mean DBH does not necessarily lead to an increase in carbon storage if species abundance is not constant (Larsary et al. 2021). The slight negative impact of minimum DBH may indicate that small‐diameter trees have a limited contribution to ACS. As the second most important biological factor, Hmean has a positive effect on ACS (SHAP value of 0.437), while Hmax has a significant negative impact on ACS (SHAP value of −8.184). This discrepancy may be attributed to the limitation of the model in simulating the relationship between ACS growth and tree height growth during the prediction process. It seems counterintuitive that the growth rate of ACS would exceed that of tree height, yet this result raises questions about the validity of the prediction.

In terms of biodiversity, although tree abundance has a relatively small impact (with a SHAP value of 0.455), it still contributes positively to ACS prediction. However, the Pielou evenness index has a significant negative effect on ACS (with a SHAP value of −1.684), indicating that an even distribution of tree species individuals is not conducive to the accumulation of forest ACS. In real forest ecosystems, niche differentiation leads to an uneven distribution of species individuals, with species that are more competitive for resources having higher population numbers and thus accumulating more ACS (Xu et al. 2012). Species richness (SR) reflects the complexity and stability of the forest ecosystem, and we found a positive correlation between SR and ACS. Higher species richness typically implies a greater abundance of niches and higher functional redundancy (Wang et al. 2022), which helps to enhance the resilience and recovery of the forest ecosystem, thereby contributing to the accumulation of ACS.

Among the environmental factors, altitude has the strongest negative impact (with a SHAP value of −6.412), indicating that as altitude increases, the ACS of forests tends to decrease (Wen et al. 2022). This finding is consistent with the ecological theory of altitudinal gradients, where increasing altitude typically brings climate changes, like lower temperatures and reduced precipitation, which negatively impact vegetation growth and carbon accumulation (Lu et al. 2023). Second, slope, as another significant positive influencing factor, indirectly affects tree growth and distribution, and thus the distribution of forest carbon storage, by influencing soil drainage, light conditions, and soil erosion (McEwan et al. 2011). Both convexity and slope aspect exert negative effects on carbon storage. Slope aspect primarily influences light conditions and temperature distribution, which in turn affect vegetation growth and distribution. Convexity, a metric describing the complexity of surface morphology or canopy shape, has a negative impact, suggesting that under complex or irregular surface morphologies, forest growth and carbon accumulation may be constrained to some extent (McEwan et al. 2011). This may be related to differences in local soil conditions, light distribution, and water use efficiency among tree species within the forest ecosystem.

It is important to acknowledge that, despite significant findings, this study has limitations. Firstly, while the south subtropical forests in Dinghushan Nature Reserve exhibit remarkable diversity in vegetation composition and encompass various forest types that are rarely found in other regions at the same latitude worldwide, with their community structure and species composition characteristics being representative of south subtropical forest communities in southern China, the sample size of five 1 hm^2^ forests may limit the generalizability of the model prediction results. Secondly, the performance of the models is constrained by specific environmental conditions and data quality. Notably, the lack of comprehensive climate data represents an additional limitation, as climate factors are known to significantly influence forest dynamics and ecosystem processes. Future research can address these limitations by expanding the sample size and more comprehensively considering different biotic and abiotic factors. Additionally, while this study focuses on different types of subtropical forests in southern China and the models demonstrate applicability in this region, further validation is needed to determine their suitability for forests in other climatic zones.

## 5 Conclusion

Based on actual survey data from different forest types in subtropical southern China, this study predicts and compares the performance of four machine learning models—SVM, RF, MLP, and XGB—for estimating forest carbon storage. The study aims to reveal the potential advantages and limitations of these models in estimating ACS in subtropical forests of southern China. The results indicate that the XGB model exhibits the best prediction performance and strongest adaptability for estimating ACS in subtropical southern China forests, followed by the MLP model. In contrast, the SVM and RF models demonstrate relatively poorer prediction performance. For the best‐performing XGB model, a deeper exploration into the impact of different feature factors on its ACS predictions was conducted, revealing varying roles played by DBH, tree height, species diversity, and environmental factors. The DBH feature plays a pivotal role in predicting ACS, particularly the maximum DBH, whose significant positive impact underlines the dominance of large trees in forest ACS. However, the negative effects of the mean and minimum DBH values reveal the complex influence of DBH distribution on ACS, indicating that trees of different DBH classes play distinct roles in carbon accumulation. Regarding tree height characteristics, the average tree height exerts a slight positive influence on ACS, while the significant negative impact of maximum tree height reveals the intricate relationship between tree height and carbon accumulation, which may be regulated by multiple factors such as resource competition and ecological conditions. Among environmental factors, the strong negative effect of altitude suggests that climatic conditions deteriorate with increasing altitude, posing adverse conditions for forest carbon accumulation. In contrast, slope exerts a significant positive effect on ACS by optimizing soil drainage, light conditions, and other factors. Research on biodiversity reveals its complex role in the carbon cycle. The positive contribution of abundance to ACS indicates that an increase in species number facilitates carbon accumulation. Future research directions could include comparing more models, considering a wider range of ecological and environmental factors, and further improving model performance. These findings have identified the most suitable machine learning model for accurately assessing ACS in subtropical forests in southern China, while also providing effective methods for precise estimation of forest ACS.

## Author Contributions

**Jiarun Liu:** data curation, resources (equal), software (equal), visualization, writing – original draft, writing – review and editing (equal). **Zihang Yang:** data curation, software (equal), writing – review and editing (equal). **Lin Li:** conceptualization, investigation, project administration, supervision, visualization, writing – review and editing (equal). **Xiaoxue Chu:** draw diagrams. **Shiguang Wei:** funding acquisition, resources, conceptualization, review and editing, investigation. **Juyu Lian:** investigation, data support.

## Conflicts of Interest

The authors declare no conflicts of interest.

## Supporting information


**Table S1.** Descriptive statistics of variables for stepwise regression analysis.

Data S1.

## Data Availability

The data that support the findings of this study are available in the Additional files of this article.
