# Supplementary material for: Predicting Aboveground Carbon Storage in Different Types of Forests in South Subtropical Regions Using Machine Learning Models
Source: Ecol Evol. 2025 May 26;15(5):e71499. doi: 10.1002/ece3.71499 (PMC12105939; doi:10.1002/ece3.71499)
Supplement: Supplementary file 1 — Table S1. Descriptive statistics of variables for stepwise regression analysis. [file ECE3-15-e71499-s002.docx]

**Appendix**

Table S1 Descriptive Statistics of Variables for Stepwise Regression Analysis

|  | Variable | max | | min | | mean | SD |
| --- | --- | --- | --- | --- | --- | --- | --- |
|  | elevation | 655.750 | 40.863 | | 212.769 | | 211.577 |
| Topographic factors | aspect | 359.730 | 0.222 | | 180.376 | | 153.208 |
|  | convexity | 25.455 | 0.004 | | -0.216 | | 6.719 |
|  | slope | 359.556 | 0.222 | | 45.019 | | 83.601 |
|  | DBHmean | 21.823 | 2.131 | | 6.861 | | 2.252 |
|  | Hmean | 9.912 | 1.022 | | 5.677 | | 1.064 |
| Species characteristics | DBHmax | 187.000 | 6.500 | | 31.775 | | 19.739 |
|  | DBHmin | 4.400 | 0.500 | | 1.516 | | 0.531 |
|  | Hmax | 99.000 | 5.500 | | 15.467 | | 7.808 |
|  | Hmin | 5.000 | 0.500 | | 2.113 | | 0.683 |
|  | Abundance | 70.000 | 4.000 | | 25.958 | | 12.444 |
|  | Shannon indices | 2.916 | 0.410 | | 2.051 | | 0.424 |
| diversity index | Simpson indices | 0.933 | 0.104 | | 0.800 | | 0.112 |
|  | SR species richness | 26.000 | 1.000 | | 12.727 | | 4.186 |
|  | Pielou indices | 0.976 | 0.095 | | 0.819 | | 0.103 |
|  | NDVI | 0.722 | 0.031 | | 0.363 | | 0.172 |
|  | NDPI | 0.678 | 0.119 | | 0.573 | | 0.121 |
| remote sensing data | NDWI | 0.170 | -0.681 | | -0.338 | | 0.170 |
|  | OSAVI | 0.722 | 0.031 | | 0.363 | | 0.172 |
|  | EVI | 1.000 | 0.008 | | 0.803 | | 0.443 |
|  | GNDVI | 0.681 | 0.020 | | 0.339 | | 0.169 |
